# FLP-18 Functions through the G-Protein-Coupled Receptors NPR-1 and NPR-4 to Modulate Reversal Length in *Caenorhabditis elegans*

**DOI:** 10.1523/JNEUROSCI.1955-17.2018

**Published:** 2018-05-16

**Authors:** Ashwani Bhardwaj, Saurabh Thapliyal, Yogesh Dahiya, Kavita Babu

**Affiliations:** Department of Biological Sciences, Indian Institute of Science Education and Research, Manauli PO 140306, Punjab, India

**Keywords:** *flp-18*, *npr-1*, *npr-4*, reversal length

## Abstract

Animal behavior is critically dependent on the activity of neuropeptides. Reversals, one of the most conspicuous behaviors in *Caenorhabditis elegans*, plays an important role in determining the navigation strategy of the animal. Our experiments on hermaphrodite *C. elegans* show the involvement of a neuropeptide FLP-18 in modulating reversal length in these hermaphrodites. We show that FLP-18 controls the reversal length by regulating the activity of AVA interneurons through the G-protein-coupled neuropeptide receptors, NPR-4 and NPR-1. We go on to show that the site of action of these receptors is the AVA interneuron for NPR-4 and the ASE sensory neurons for NPR-1. We further show that mutants in the neuropeptide, *flp-18*, and its receptors show increased reversal lengths. Consistent with the behavioral data, calcium levels in the AVA neuron of freely reversing *C. elegans* were significantly higher and persisted for longer durations in *flp-18*, *npr-1*, *npr-4*, and *npr-1 npr-4* genetic backgrounds compared with wild-type control animals. Finally, we show that increasing FLP-18 levels through genetic and physiological manipulations causes shorter reversal lengths. Together, our analysis suggests that the FLP-18/NPR-1/NPR-4 signaling is a pivotal point in the regulation of reversal length under varied genetic and environmental conditions.

**SIGNIFICANCE STATEMENT** In this study, we elucidate the circuit and molecular machinery required for normal reversal behavior in hermaphrodite *Caenorhabditis elegans*. We delineate the circuit and the neuropeptide receptors required for maintaining reversal length in *C. elegans*. Our work sheds light on the importance of a single neuropeptide, FLP-18, and how change in levels in this one peptide could allow the animal to change the length of its reversal, thereby modulating how the *C. elegans* explores its environment. We also go on to show that FLP-18 functions to maintain reversal length through the neuropeptide receptors NPR-4 and NPR-1. Our study will allow for a better understanding of the complete repertoire of behaviors shown by freely moving animals as they explore their environment.

## Introduction

Neuropeptides are used by neurons to communicate with each other and to modulate behavior. They modulate synaptic activity through synaptic and extrasynaptic neuropeptide receptors. Neurons generally signal through only one neurotransmitter, but they can secrete multiple neuropeptides. One difficulty in studying neuropeptide function is that a single neuropeptide can signal through multiple receptors (Li and Kim, 2008).

Locomotion is the basis for most behaviors in *Caenorhabditis elegans*, including feeding, mating, sleeping, avoidance, and escape behaviors. The animal's movement is mediated by multiple sets of motor neurons along the ventral nerve cord innervating the dorsal and ventral body-wall muscles (White et al., 1976). Muscle contractions are mediated by cholinergic A- and B-type motor neurons that innervate these muscles (White et al., 1986; Alfonso et al., 1993), whereas muscle relaxations are mediated by the GABAergic D-type motor neurons (White et al., 1986; McIntire et al., 1993a, b). The multiple sets of motor neurons that innervate the body-wall muscles receive synaptic inputs from interneurons, which are in turn stimulated by sensory neurons (Goodman, 2006). Together, these neurons coordinate the activity of the ventral and dorsal muscles to generate the sinusoidal waves for forward locomotion, interspersed by short bouts of backward movement or reversals.

Reversals are critical determinants of the *C. elegans* navigation strategy as they increase the probability of change in direction. Reversal behavior can be parsed into two components: reversal initiation, which determines the frequency of reversals; and reversal termination, which determines the reversal length. Shorter reversals allow for change in directions at angles <90 degrees, whereas longer reversals are almost always followed by ω turns that lead to an ∼180 degree change in direction of locomotion. Laser ablation of the AVA command interneuron completely eliminated longer reversals, but the animal could still initiate reversals, suggesting that reversal initiation (frequency) and regulation of reversal length might have a different cellular basis (Gray et al., 2005).

Although a number of studies have highlighted the neurons, molecules, and circuits that are required for the reversal behavior in *C. elegans* (Chalfie et al., 1985; Kaplan and Horvitz, 1993; Hart et al., 1995; Maricq et al., 1995; Y. Zheng et al., 1999; Brockie et al., 2001; Zhao et al., 2003; Alkema et al., 2005; Gray et al., 2005; Cohen et al., 2009; Piggott et al., 2011; Campbell et al., 2016), most of these studies were focused on the reversal frequency. The role of neurons in regulating reversal length has to date been elaborated in a single previous study involving laser ablation of neurons (Gray et al., 2005). A very recent study has shown that the AVA interneurons control reversal state by affecting the motor neurons and that the P-/Q-type calcium channel UNC-2 is required for maintaining the duration of reversals through its effect on motor neuron oscillations. The authors further showed that enhancing UNC-2 activity shows an increase in reversal duration and velocity (Gao et al., 2018). However, although the neurons required for maintaining reversal length and the role of UNC-2 in this process have been studied, the molecular mechanisms that regulate the length of reversals remain largely unknown.

Studies have shown that the neuropeptide FLP-18 is required for modulating spontaneous reversal frequency and swimming rates in *C. elegans* (Kim and Li, 2004; Cohen et al., 2009; Y.J. Chang et al., 2015). However, the effects of FLP-18 on reversal length have not been studied. We show that FLP-18 controls reversal length by regulating the function of AVA interneurons and ASE sensory neurons through the G-protein-coupled neuropeptide receptors NPR-4 and NPR-1, respectively. Our experiments suggest a possible correlation between increased reversal length and increased activity of the AVA neuron in *flp-18*, *npr-1*, and *npr-4* genetic backgrounds in freely moving animals. We further show that FLP-18 expression is regulated by the transcription factor CREB1/CRH-1 as well as by starvation and that both loss of *creb1/crh-1* and starvation cause decrease in reversal length through the FLP-18 pathway. This indicates that FLP-18 could act as a behavioral switch from local search to global search behavior under different genetic conditions as well as during starvation. Our work identifies the FLP-18/NPR-1/4 signaling pathway as a pivotal point in defining an aspect of *C. elegans* locomotion under varied environmental and genetic conditions.

## Materials and Methods

### 

#### 

##### Strains.

All the strains used in this work were grown at 20°C under standard conditions using nematode growth medium (NGM) plates. The N2 strain was used as the reference wild-type (WT) strain. The list of strains used in this study is given in Table 1. All experiments were performed with hermaphrodite *C. elegans*.

**Table 1.** List of strains used in this study

Strain	Genotype	Comment	Figure(s)
BAB1541	*flp-18* (CGC strain VC2016)	From CGC (outcrossed 3×)	1, 1-1, and 4
BAB1542	*npr-1* (CGC strain RB1330)	From CGC (outcrossed 3×)	1 and 1-1
BAB1543	*npr-4* (Mittani strain *tm1782*)	From NBRP (outcrossed 3×)	1 and 1-1
BAB1544	*npr-5* (CGC strain CX14394)	From CGC (outcrossed 3×)	1-1
BAB1501	*npr-1 npr-4*		1, 1-1, and 4
BAB1502	*flp-18 npr-1 npr-4*		1 and 1-1
BAB765	*crh-1* (CGC strain YT17)	From CGC (outcrossed 3×)	3
BAB727	*crh-1; Prab-3::*CRH-1f (*indEx719*)		3
BAB1503	*flp-18;* P*flp-18::*FLP-18::*sl2*::GFP		3
BAB1504	*npr-1 npr-4;* P*flp-18::*NPR-4a (*indEx501*)		1
BAB1505	*npr-1 npr-4;* P*rig-3::*NPR-4a (*indEx504*)		1
BAB1506	*npr-1 npr-4;* P*unc-4::*NPR-1 (*indEx502*)		1
BAB1517	*npr-1 npr-4;* P*sra-6::*CHOP-2(H134R):: mCherry *+* P*osm-10::G-CaMP +* P*unc-122::* mCherry*;* P*rig-3::*NPR4a (*indEx504*)	*npr-4* rescue in AVA	1
AX1444	P*flp-18::*FLP-18::*sl2*::GFP	Cohen et al. (2009)	3, 4
BAB1509	*crh-1;* P*flp-18::*FLP-18::*sl2*::GFP		3
BAB1510	*crh-1;* P*flp-18::*FLP-18::*sl2*::GFP*;* P*rab-3::*CRH-1f		3
BAB1511	*crh-1;* P*flp-18::*FLP-18::*sl2*::GFP*;* P*flp-18::CRH-1f* (*indEx505*)		3
BAB1512	P*sra-6::*CHOP-2(H134R)::mCherry *+* P*osm-10::G-CaMP +* P*unc-122::*mCherry	Guo et al. (2009)	1, 3
BAB1513	*flp-18;* P*sra-6::*CHOP-2(H134R)::mCherry *+* P*osm-10::G-CaMP +* P*unc-122::*mCherry		1
BAB1514	*crh-1;* P*sra-6::*CHOP-2(H134R):: mCherry *+* P*osm-10::G-CaMP +* P*unc-122::* mCherry		3
BAB1515	*npr-1 npr-4;* P*sra-6::*CHOP-2(H134R):: mCherry *+* P*osm-10::G-CaMP +* P*unc-122::* mCherry		1
BAB1516	*flp-18 npr-1 npr-4;* P*sra-6::*CHOP-2(H134R):: mCherry *+* P*osm-10::G-CaMP +* P*unc-122::* mCherry		1
BAB1519	*crh-1;Psra-6::*CHOP-2(H134R):: mCherry *+* P*osm-10::G-CaMP +* P*unc-122::* mCherry*;* P*flp-18::*CRH-1f (*indEx505*)	*crh-1* rescue in FLP-18-expressing neurons	3
CX15380	P*rig-3::*GCaMP5	Larsch et al. (2013)	2
BAB1523	*flp-18;* P*rig-3::*GCaMP5		2
BAB1524	*npr-1;* P*rig-3::*GCaMP5		2
BAB1525	*npr-4;* P*rig-3::*GCaMP5		2
BAB1526	*npr-1 npr-4;* P*rig-3::*GCaMP5		2
BAB1527	*crh-1; flp-18*		3
BAB1528	*npr-1 npr-4; Punc-30::*NPR1::*sl2::*wrmScarlet		1 and 1-1
BAB1529	*npr-1 npr-4; Pgpa-3::*NPR1::*sl2::*wrmScarlet; *Punc122::*GFP		1 and 1-1
BAB1530	*npr-1 npr-4; Pflp-5::*NPR1*::sl2::wrmScarlet; Punc122::*GFP		1 and 1-1
BAB1531	*npr-1 npr-4; Pgcy-5::*NPR1*::sl2::wrmScarlet; Punc122::*GFP		1 and 1-1
BAB1532	*npr-1 npr-4; Pgcy-7::*NPR1*::sl2::wrmScarlet; Punc122::*GFP		1 and 1-1
BAB1533	*npr-1 npr-4; Pgcy-5::*NPR1*::sl2::wrmScarlet; Pgcy-7::*NPR1*::sl2::wrmScarlet; Punc-122::*GFP		1
BAB1534	*npr-1 npr-4; Prig-3::*NPR4*::sl2::wrmScarlet*		1 and 1-1
BAB1535	*npr-1 npr-4; Prig-3::*GCaMP5; *Prig-3::*NPR4*::sl2::wrmScarlet*		2
BAB1536	*npr-1 npr-4; Prig-3::*GCaMP5; *Pgcy-5::*NPR1*::sl2::wrmScarlet; Pgcy-7::*NPR1*::sl2::wrmScarlet*		2
BAB1538	*crh-1; Pflp-18::*FLP-18::*sl2*::GFP*; Pflp-18::CRH-1f; Pnmr-1::*mCherry*; Pttx-3::*mCherry		2
BAB1539	*Pflp-18::*FLP-18::*sl2*::GFP*; Pnmr-1::*mCherry*; Pttx-3::*mCherry		2
BAB1540	*crh-1; Pflp-18::*FLP-18::*sl2*::GFP*; Pnmr-1::*mCherry*; Pttx-3::*mCherry		2

##### Molecular biology and transgenic lines.

The promoters used for this study include *flp-18* (4.3 kb), *rig-3* (3 kb), *unc-30* (3 kb), *gcy-5* (3 kb), *gcy-7* (1.2 kb), *gpa-3*(5.8 kb), and *unc-4* (2 kb) all of which have been previously described (Eastman et al., 1999; Lickteig et al., 2001; Rogers et al., 2003; Feinberg et al., 2008). These promoters were cloned into pPD95.75 or pPD49.26 along with the cDNA of the gene of interest expressed downstream of the promoter. The transgenic lines were prepared by microinjecting plasmids of interest as described previously (Mello et al., 1991; Mello and Fire, 1995; Lickteig et al., 2001).

A list of all the primers used to make constructs and genotype the *C. elegans* in this work is given in Table 2, and the plasmids used in this work are indicated in Table 3.

**Table 2.** List of primers used in this study

Primer code	Sequence	Comment	Gene
AB113	AGGACGGAAATTACCTGTGC	Genotyping forward external	*flp-18*
AB114	GCTTCGGGAAACGCTCATAT	Genotyping reverse internal	*flp-18*
AB115	TTATTCTTTCTTGTCGGGGCC	Genotyping reverse external	*flp-18*
AB116	ACCTGTCACTTTTACGCCGG	Genotyping forward external	*npr-1*
AB117	TGATTTCGTTCCAGTTGAACG	Genotyping reverse internal	*npr-1*
AB118	GAACCTTCACTTCTCCTGTG	Genotyping reverse external	*npr-1*
AB119	AGCTGTTGTCTCCTTCCAGG	Genotyping forward external	*npr-4*
AB120	CGATTTCCGATGAGGAAACC	Genotyping reverse internal	*npr-4*
AB121	CACAGCTTCTAATAGGAAAGGG	Genotyping reverse external	*npr-4*
AB122	GCACGACGAACTGCAAATTT	Genotyping forward external	*npr-5*
AB123	TCCTTGAGTTTTCTGGGATG	Genotyping reverse internal	*npr-5*
AB124	AGGCATTTTTGGAAACGGCG	Genotyping reverse external	*npr-5*
AB64	ACGCGTCGACAATGCTGTCCCGGAACTGGGAT	Cloning forward SalI site	P*unc-4*
AB65	CCCCCCGGGAAAGAAGAACCCACTTCGGCTC	Cloning reverse XmaI site	P*unc-4*
AB108	ACGCGTCGAC TCTGTCACATACTGCTCGAA	Cloning forward SalI site	P*flp-18*
AB109	CCCCCCGGGGTTGCTGTCTAACCCTGAAA	Cloning reverse XmaI site	P*flp-18*
AB134	CTAGCTAGCATGAATGGCTCCGATTGTCT	Cloning forward NheI site	*npr-4a* cDNA
AB136	CGGGGTACCTTAGAAAGAAGCCTTCCTTGGT	Cloning reverse KpnI site	*npr-4a* cDNA
AB130	CTAGCTAGCATGGAAGTTGAAAATTTTACCGACTG	Cloning forward NheI site	*npr-1* cDNA
AB131	CGGGGTACCTCAGACTAGCGTGTCGTTGA	Cloning forward NheI site	*npr-1* cDNA
AB139	GCGTCGACAAGTGACACCACGCTCACA	Cloning forward SalI site	P*rig-3*
AB140	CCCCCCGGGAGCTGTGAAATTTTTAGGCAGT	Cloning reverse XmaI site	P*rig-3*
AB192	ACATGCATGCCGATTTAAACCTAAAACAGTTGAAAG	Cloning forward SphI	P*flp-5*
AB193	CCCCCCGGGGTAAAAGGCGGGTGCTGTC	Cloning reverse XmaI	P*flp-5*
AB209	ACATGCATGCACAAAGTTTTTAAAAAGTTGTTGATCGG	Cloning forward SphI	P*gpa-3*
AB210	CCCCCCGGGGAAGCACAACTCTAAAAAGCCCA	Cloning reverse XmaI	P*gpa-3*
AB216	ACATGCATGCCGATTGACATTGGTCTTACATTTTGAC	Cloning forward SphI	P*gcy-5*
AB217	CCCCCCGGGATTGAAATTCTACTACTTCTGGGGG	Cloning reverse XmaI	P*gcy-5*
AB219	ACATGCATGCAATAAAAAGCAAAACAGCGAGTCAA	Cloning forward SphI	P*gcy-7*
AB220	CCCCCCGGGGATTATTTTCTTATGCTAAACTGGCAGA	Cloning reverse XmaI	P*gcy-7*
YD169	ATTAGCTAGCATGGAGTCACTGGTTTTCAATGG	Cloning forward NheI site	*crh-1f* cDNA
YD164	ATTACCATGGTCACATTCCGTCCTTTTCCTTTC	Cloning reverse NcoI site	*crh-1f* cDNA
YD157	TGGAAGGAGGAGGAGATGGAAA	Genotyping forward external	*crh-1*
YD158	GCAGTACAGCTCTTTCAGCGTT	Genotyping Forward Internal	*crh-1*
YD159	AATTCGGCACAACGGACTGG	Genotyping reverse external	*crh-1*

**Table 3.** List of plasmids used in this study

Serial no.	Plasmid no.	Plasmid
1	pBAB501	P*flp-18::*NPR-4a
2	pBAB504	P*rig-3::*NPR-4a
3	pBAB503	P*unc-25::*NPR-1
4	pBAB502	P*unc-4::*NPR-1
5	pBAB719	P*rab-3::*CRH1f
6	pBAB505	P*flp-18::*CRH1f
7	pBAB509	P*unc-30::*NPR-1::*sl2::*wrmScarlet
8	pBAB510	P*flp-5::*NPR-1::*sl2::*wrmScarlet
9	pBAB511	P*gpa-3::*NPR-1::*sl2::*wrmScarlet
10	pBAB512	P*gcy-5::*NPR-1::*sl2::*wrmScarlet
11	pBAB513	P*gcy-7::*NPR-1::*sl2::*wrmScarlet
12	pBAB513	P*gcy-7::*NPR-4::*sl2::*wrmScarlet

##### Behavioral assays.

Well-fed young adult animals were used to conduct all the behavioral studies, except for the starvation experiments. The reversal assays were performed after transferring the *C. elegans* to food-free NGM plates. Observation time started 1 min after transfer. The *C. elegans* were transferred using halocarbon oil or eyelash picks to avoid any injury to the animal. Spontaneous reversals were scored for 5 min as previously described (Zhao et al., 2003). The reversal length is reported throughout this work as body bends per reversal. We defined one body bend as equal to one-third the length of the *C. elegans*. For all reversal assays, a 5 min video was made and the number of body bends per reversal calculated for every reversal made during the 5 min that was recorded, the number of body bends was then averaged over every reversal made during the recording and was plotted as a single dot in the scatter plot. More than 20 such recordings were analyzed for each genotype. The results were plotted as mean ± SEM and evaluated using the standard Student's *t* test.

For the starvation assays, animals were starved for 24 h at the L4 stage (54 h including hatching time) on peptone free plates (to avoid contamination). After 24 h, *C. elegans* were assayed for reversal behavior. Again, reversal length was determined for each spontaneous reversal.

##### Optogenetic assays.

To stimulate a specific neuron, channelrhodopsin-2 (ChR2) was used as previously described (Husson et al., 2013). The *sra-6* promoter was used to drive ASH neuron-specific expression of ChR2 as previously reported in the SRS85 strain (*sraIs49* V; *lite-1* (*ce314*) X; *sraEx80*) (Guo et al., 2009). Animals expressing ChR2 were grown on NGM agar plates seeded with OP50-containing 400 μm All-trans retinal. The *C. elegans* were grown in the dark until late L4/early adult stages. The assay was performed on freshly seeded NGM plates. During the assay, ChR2 was excited by blue light (460–490 nm) sourced from an epifluorescence unit (U-HGLGPS, Olympus) attached to the Nikon SMZ2000 microscope. This experiment was performed under low illumination to avoid the preactivation of ChR2. Blue light was illuminated for 3 s, and reversal length was quantified as number of body bends in response to illumination. This assay was done with >20 animals per genotype, and each dot in the scatter plot indicates the number of body bends in a single reversal brought about by stimulation of ASH. The results were plotted as mean ± SEM and evaluated using the standard Student's *t* test. The experimenter was blind to the genotypes of the strains while performing these experiments.

##### Calcium imaging.

The genetically encoded Ca^2+^ indicator GCaMP-expressing strain P*rig-3*::GCaMP5 was used to visualize Ca^2+^ transients in the AVA command interneuron (Larsch et al., 2013). Calcium transients were recorded in freely navigating *C. elegans* as previously described (Faumont et al., 2011) using an Olympus IX73 inverted microscope fitted with the ASI-based worm-tracker. Imaging was done at 40× objective with 0.6 NA. Images were acquired through QImaging camera using the ImageJ software. Videos were recorded at 10 frames per second with 100 ms exposure.

The AVA neuron activity was analyzed according to the protocol described previously (Kerr, 2006). The analysis was done using the FIJI ImageJ software. The ROI was drawn as a 25 × 25 pixel circle over the AVA cell body-expressing GCaMP. The measured average pixel value from the ROI, F_meas_, includes fluorescence from sample and background fluorescence (F_bkg_). Then the fluorescence (F) from the given ROI was estimated by subtracting background fluorescence from the measured fluorescence value (i.e., F = F_meas_ − F_bkg_). The fluorescence value was estimated for each frame after 100 ms by manual repositioning of the ROI. Calcium transients were plotted as ΔF/F_o_, where ΔF is the change in the fluorescence value (F) from its baseline fluorescence (F_o_). Calcium levels were estimated as ΔF/F_o_ max, which indicated the maximum ΔF/F_o_ value for each animal. Calcium-raising duration in AVA was calculated from the frame when the animal initiated the reversal to the end of the reversal. The standard Student's *t* test was used to compare the ΔF/F_o,_ duration of AVA and the calcium-raising duration (Δt) values between the WT and mutant strains.

##### Microscopy.

For all fluorescence confocal microscopy-based experiments, except the experiments involving starvation assays, late L4/early adult *C. elegans* were imaged. The animals were paralyzed using 2,3-butanedione monoxime (30 mg/ml) for imaging p*flp-18*::FLP-18::*sl2*::GFP in the neurons of the head region (Cohen et al., 2009). Images were acquired using a Leica TCS SP8 confocal microscope. Imaging of starvation experiments was done 24 h following the L4 stage. The fluorescence intensity from the ROI was quantified as the integrated intensity using ImageJ. The data are expressed as mean ± SEM. To perform neuron-specific imaging, the neuron of interest was identified by expressing the mCherry marker under a neuron-specific promoter. The *Pnmr-1*::mCherry and *Pttx*-3::mcherry promoters were used as markers to identify AVA, RIM, and AIY. GFP fluorescence quantification of P*flp-18*::FLP-18::*sl2*::GFP was performed from a single neuron for each animal using ImageJ. Imaging results were evaluated using standard Student's *t* test.

##### Statistical analysis.

The Student's *t* test (unpaired *t* test with Welch's correction) was performed using Prism 6 (GraphPad). All the statistical values for unpaired *t* test were provided as (*p*, *t*, *df*). The level of significance was set as *p* < 0.05

## Results

### FLP-18 functions to control reversal length through the NPR-4 receptor in AVA interneurons and the NPR-1 receptor in ASE sensory neurons

A neuropeptide of the FMRFamide-like family, FLP-18 has been implicated in multiple behaviors in *C. elegans* (Kim and Li, 2004; Cohen et al., 2009; Y. J. Chang et al., 2015). FLP-18 and its receptors have been reported to be expressed in the neural circuitry involved in backward locomotion, and specifically in the AVA command interneuron (Rogers et al., 2003; Kim and Li, 2004; Li and Kim, 2014).

**Figure 1.**
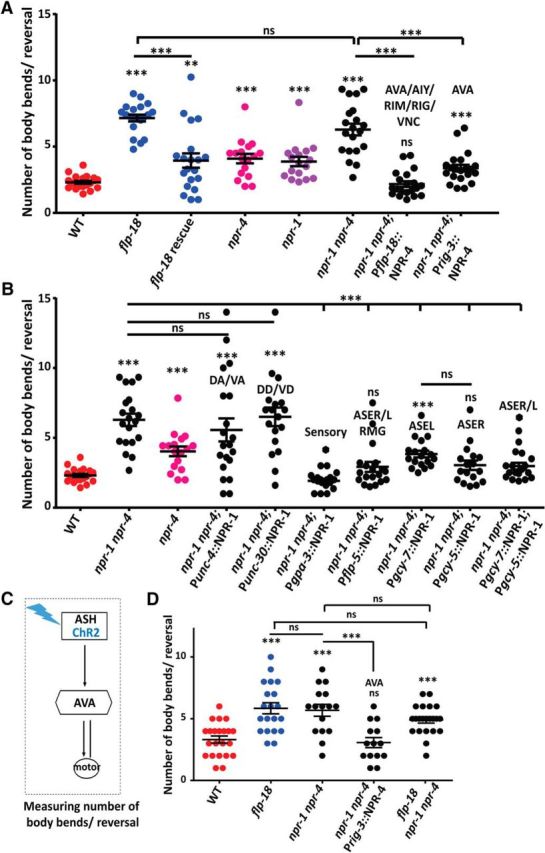
Mutants in *flp-18* show increased body bends per reversal. ***A***, Body bends per reversal in WT, *flp-18*, *npr-1*, *npr-4*, *npr-1 npr-4*, and NPR-4 rescue lines (*n* = 20). ***B***, Body bends per reversal in WT, *npr-4*, *npr-1 npr-4*, and NPR-1 rescue lines (*n* = 17–20). ***C***, ASH activation using ChR2. ***D***, Body bends per reversal upon ChR2-based activation of ASH interneurons in WT, *flp-18*, *npr-1 npr-4*, NPR-4 rescue line, and the *flp-18 npr-1 npr-4* triple mutants (*n* = 20). Error bars indicate SE. See also Figure 1-1. **p < 0.01, ***p < 0.001.

We were interested in further understanding the role of FLP-18 in the modulation of the exploratory behavior of *C. elegans*. Similar to what was previously published by Cohen et al. (2009), we found that *flp-18* mutants showed decreased reversal frequency compared with WT animals (*p* = 0.0052, *t* = 2.981, df = 34.8; Fig. 1-1*A*). We also observed longer reversals: that is, increased body bends per reversal in *flp-18*-null mutants, compared with WT control animals (*p* < 0.0001, *t* = 17.36, df = 26.45; Fig. 1*A*; https://www.amazon.com/clouddrive/share/2LD28GVXT2kWSxEOixgxjlI9TfxKTpXZxtkXbquvzXR and https://www.amazon.com/clouddrive/share/KVIPVONyLYu7A69KkIILW9ov0tKyEQgO2JT5zHlCGBa). This observation allowed us to postulate that FLP-18 could be modulating the reversal circuitry required to regulate the length of reversals. Further, expressing FLP-18::*sl2*::GFP under its own promoter partially rescued the increased reverse body bends seen in the mutants (*p* = 0.0075, *t* = 2.963, df = 20.71; Fig. 1*A*). Previous reports have indicated that the FLP-18::*sl2*::GFP line shows abnormal movement behaviors (Cohen et al., 2009), which could account for the partial rescue of the *flp-18* mutant phenotype (*p* < 0.0001, *t* = 5.367, df = 27.01). To affirm that *flp-18* does not show obvious movement defects, we compared the frequency of forward body bends made by the *flp-18* mutants with WT control animals and found this frequency to be similar to WT levels (*p* = 0.413, *t* = 0.832, df = 26.29; Fig. 1-1*B*).

10.1523/JNEUROSCI.1955-17.2018.f1-1 Figure 1-1 (A) Graph of number of reversals per five minutes in WT animals in comparison to *flp-18* mutant *C. elegans* (n=20).(B) Graph of forward body bends per 30s in WT and *flp-18* mutants (n=20).(C) Graph of number of body bends per reversal in WT and *npr-5* mutant animals (n=16-20). (D) Indicates images of NPR-4 and NPR-1 under specific promoters. The promoter is upstream of *npr-4* or *npr-1* cDNA and the *sl2* sequence tagged to wrmScarlet (scale bar; 20μm). (E) Graph of body bends per reversal in wild-type (WT), *flp-18, npr-1 npr-4* double mutants and *flp-18 npr-1 npr-4* triple mutants (n=20).The error bars indicate standard errors in all figures. Download Figure 1-1, TIF file

We next wanted to delineate the receptors through which FLP-18 could be functioning to mediate reversal length. Previous work has shown that FLP-18 has high binding affinity for the receptors NPR-1, NPR-4, NPR-5, NPR-10, and NPR-11 (Li and Kim, 2014). AVA ablation eliminates longer reversals in both off and on food; therefore, we reasoned that FLP-18 was likely modulating reversal length at the interneuron level (Gray et al., 2005). Sensory neurons give cues to activate the command interneuron AVA, which causes activation of reversals (Piggott et al., 2011; Pirri and Alkema, 2012; Gordus et al., 2015). We hypothesized that FLP-18 could be functioning through the command interneuron AVA or the upstream sensory neurons to regulate reversal length. The expression pattern of NPR-10 has not been well documented; however, NPR-1 is expressed in sensory and motor neurons and NPR-4 is largely expressed in the AVA command interneurons, whereas NPR-5 and NPR-11 are expressed mainly in sensory neurons (de Bono and Bargmann, 1998; Wang and Wadsworth, 2002; Cohen et al., 2009; Chalasani et al., 2010). Furthermore, FLP-18 has been shown to function through NPR-1 or NPR-4 receptors for multiple behavioral processes (Rogers et al., 2003; Cohen et al., 2009; Frooninckx et al., 2012; Choi et al., 2013; Stawicki et al., 2013; Nagy et al., 2014; J. Luo et al., 2015). We wanted to test whether FLP-18 controls reversal length through the NPR-1 and NPR-4 receptors. We started with observing the reversal length in the *npr-1* and *npr-4* mutants. Both the mutants showed significant increase in body bends per reversal compared with WT animals: WT versus *npr-1* (*p* = 0.0004, *t* = 4.257, df = 19.44), and WT versus *npr-4* (*p* = 0.0001, *t* = 4.766, df = 19.32; Fig. 1*A*). We found that there was a significant increase in the number of body bends per reversal in the *npr-1 npr-4* double mutants compared with either of the single mutants: *npr-1* versus *npr-1 npr-4* (*p* = 0.0001, *t* = 4.283, df = 34.32), *npr-4* versus *npr-1 npr-4* (*p* = 0.0005, *t* = 3.867, df = 34.48). Further, the double mutants showed a phenotype that was very similar to the *flp-18* mutants (*p* = 0.0982, *t* = 1.706, df = 30.41; Fig. 1*A*; https://www.amazon.com/clouddrive/share/CbcUUwOTQhF3btDNGG71l2V8POJeGyWpRRDALaZXIn2). We also tested the body bends per reversal in *npr-5* mutant animals to understand the role of other FLP-18 receptors in this process. We did not see an obvious difference in reversal lengths in these mutants compared with WT animals (*p* = 0.417, *t* = 0.829, df = 19.98; Fig. 1-1*C*).

We next wanted to identify the site of action of NPR-1 and NPR-4 receptors that would allow them to transduce the signal from FLP-18 to control reversal length. The command interneurons AVA, AVE, and AVD are required to promote reversal behavior in *C. elegans* (Chalfie et al., 1985; de Bono and Maricq, 2005; Gray et al., 2005; Piggott et al., 2011). Of these command interneurons, FLP-18 is expressed in AVA (Rogers et al., 2003; Kim and Li, 2004), which also expresses the receptor NPR-4 (Cohen et al., 2009). We thought that it was possible that NPR-4 could function in the AVA interneuron to transduce the signal through FLP-18. To test this, we performed transgenic rescue experiments for NPR-4 under the *flp-18* promoter in the *npr-1 npr-4* double mutants. We found that this rescued the *npr-4* phenotype in the *npr-1 npr-4* double mutants (*p* < 0.0001, *t* = 8.398, df = 27.49; Fig. 1*A*). We further found that the rescued animals showed a reversal length very similar to that of WT animals (*p* = 0.5856, *t* = 0.5513, df = 29.13). Because FLP-18 is expressed in multiple neurons, including AVA, RIM, AIY, and RIG, the pharyngeal neurons M2 and M3, and the ventral cord neurons (McKay et al., 2003; Rogers et al., 2003; Kim and Li, 2004), it is possible that rescue of NPR-4 with this promoter completely rescues all aspects of the *npr-1 npr-4* double mutant phenotype. To delineate a more neuron-specific role of NPR-4, we went on to do a neuron-specific rescue of NPR-4 in the AVA neuron. We expressed NPR-4 under the *rig-3* promoter using P*rig-3*::NPR-4::*sl2*::wrmScarlet, which is expressed in the AVA command interneuron (Schwarz et al., 2009) (Fig. 1-1*D*), and found that this promoter could again rescue the *npr-4* mutant phenotype in the *npr-1 npr-4* double mutants (*p* < 0.0001, *t* = 5.723, df = 30.61; Fig. 1*A*). The rescue of NPR-4 in the *npr-1 npr-4* double mutants showed a phenotype that was indistinguishable from the *npr-1* mutant animals (*p* = 0.2542, *t* = 1.162, df = 30.60), which is what would be expected if only NPR-4 is rescued in the *npr-1 npr-4* double mutant animals. These results indicate that FLP-18 could be partly functioning to maintain normal reversal body bends through the NPR-4 receptor acting in the AVA interneuron, which is one of the neurons that express FLP-18.

We also performed rescue experiments to find out where NPR-1 functions to modulate reversal length. The NPR-1 receptor has a strong expression in head sensory neurons as well as in the GABAergic D-type motor neurons, and shows a weak expression in the cholinergic A-type motor neurons (de Bono and Bargmann, 1998; Wang and Wadsworth, 2002). We first decided to rescue NPR-1 in D-type motor neurons using the *unc-30* promoter (McIntire et al., 1993a), and in the A-type motor neurons using the *unc-4* promoter (Miller and Niemeyer, 1995). We found that the expression of NPR-1 in both D-type (*p* = 0.7909 *t* = 0.2675, df = 30.03) and A-type (*p* = 0.4462 *t* = 0.7722, df = 29.04) motor neurons could not rescue the increased reversal length phenotype seen in the *npr-1 npr-4* double mutants. We next tested NPR-1 functions in sensory neurons using *gpa-3* and *flp-5* promoters. Again, we tested expression of NPR-1 under these promoters with *sl2*::wrmScarlet (Fig. 1-1*D*). The *gpa-3* promoter is expressed in many sensory neurons, including NPR-1-expressing neurons ASE, ASG, ASH, PHA, and PHB, whereas the *flp-5* promoter is largely expressed in the ray sensory neurons and the NPR-1-expressing neurons ASE and RMG. Expressing NPR-1 under both *gpa-3* and *flp-5* promoters rescued the increased reversal length phenotype seen in the *npr-1 npr-4* double mutants: *npr-1 npr-4* and *npr-1 npr-4*; P*gpa-3*::NPR-1 (*p* < 0.0001, *t* = 9.447, df = 22.73); and *npr-1 npr-4* and *npr-1 npr-4*; P*flp-5::*NPR-1 (*p* < 0.0001, *t* = 5.944, df = 35.80; Fig. 1*B*). This rescue confirmed that NPR-1 could function in sensory neurons and further indicated that NPR-1 could be modulating reversal length through the ASE sensory neurons, which are the only neurons where NPR-1 is expressed under both *gpa-3* and *flp-5* promoters. The ASE neurons have been shown to be involved in CO_2_-sensing chemotaxis to water-soluble compounds and avoidance behaviors in *C. elegans* (Bargmann and Horvitz, 1991; Sambongi et al., 1999; Pierce-Shimomura et al., 2001; Suzuki et al., 2008; Bretscher et al., 2011). To delineate a more neuron-specific role for NPR-1, we expressed NPR-1 in ASEL using the *gcy-7* promoter ASER using the *gcy-5* promoter and in both ASEL/R using both promoters (Yu et al., 1997). Expressing NPR-1 in either ASEL or ASER and in both ASEL/R significantly rescued the increased reversal length of *npr-1 npr-4* double mutant: *npr-1 npr-4* and *npr-1 npr-4*; P*gcy-7*::NPR-1 (*p* < 0.0001, *t* = 4.868, df = 29.22), *npr-1 npr-4* and *npr-1 npr-4*; P*gcy-5*::NPR-1 (*p* < 0.0001, *t* = 5.853, df = 33.96), and *npr-1 npr-4* and *npr-1 npr-4*; P*gcy-7*::NPR-1; P*gcy-5*::NPR-1 (*p* < 0.0001, *t* = 6.631, df = 28.74; Fig. 1*B*; Fig. 1-1*D*). The rescued *npr-1 npr-4* double mutants showed reversal lengths similar to that seen in the *npr-4* single mutant animals when NPR-1 was expressed only in ASEL. The reversal length was similar to WT animals when NPR-1 was expressed only in ASER or in ASER/L: WT and *npr-1 npr-4;* P*gcy-5*::NPR-1 (*p* = 0.0538, *t* = 2.051, df = 19.71); and WT and *npr-1 npr-4*; P*gcy-7*::NPR-1; P*gcy-5*::NPR-1 (*p* = 0.017, *t* = 2.557, df = 24.85; Figure 1*B*). Together, these results suggest that NPR-4 and NPR-1 act as receptors for FLP-18 in the AVA command interneuron and the ASE sensory neurons, respectively, to maintain reversal length.

Because FLP-18 appears to be functioning through NPR-1 and NPR-4, we went on to make a triple mutant of *flp-18*, *npr-1*, and *npr-4.* These mutants also showed longer reversals than WT animals (*p* = 0.027, *t* = 2.405, df = 18.23); however, the reversals were not as long as those seen in *flp-18* single mutants or *npr-1 npr-4* double mutants (Fig. 1-1*E*; https://www.amazon.com/clouddrive/share/aPkQBaOufmeJUp3Acf3KkTVctaJnM3qjST4O00WPDLr). On analyzing these mutants further, we realized that their mobility was compromised, probably due to the presence of three mutations, which are involved in multiple processes other than maintaining reversal lengths (Rogers et al., 2003; Cohen et al., 2009; Choi et al., 2013; Stawicki et al., 2013; Nagy et al., 2014; J. Luo et al., 2015).

To get a cleaner readout of the reverse body bends in the triple mutants, we decided to combine optogenetics with behavioral studies. We used a transgenic line expressing ChR2 specifically in the ASH sensory neurons using the *sra-6* promoter. We induced reversals optogenetically by stimulating the ASH neuron, which forms functional connections with the AVA interneuron (Guo et al., 2009; Lindsay et al., 2011). We noted that ASH stimulation caused WT animals to undergo reversals (illustrated in Fig. 1*C*), even though the optogenetically activated neural circuit is different from the neural circuitry that activates reversals during exploratory behavior in *C. elegans* (Gray et al., 2005; Pirri and Alkema, 2012). ChR2-mediated stimulation of the ASH neurons in *flp-18* mutants resulted in a significant increase in the number of body bends per reversal compared with WT control animals (*p* < 0.0001, *t* = 4.739, df = 32.95; Fig. 1*D*). A similar number of increased reversal body bends upon ChR2 activation were seen in *npr-1 npr-4* double mutants (*p* = 0.0003, *t* = 4.222, df = 25.48), which were significantly rescued by expressing NPR-4 in the AVA command interneuron (*p* = 0.0003, *t* = 4.132, df = 27.82). Interestingly, expressing NPR-4 in just the AVA command interneurons could completely rescue the double mutant phenotype, indicating that overexpressing NPR-4 in the double mutant was enough to reduce reversal length to WT levels (*p* = 0.629, *t* = 0.49, df = 25.21) in optogenetically induced reversals. Further, we found that the *flp-18 npr-1 npr-4* triple mutants showed a number of body bends per reversal that was similar to that seen in *flp-18* mutants (*p* = 0.078, *t* = 1.824, df = 30.31) or in *npr-1 npr-4* double mutants (*p* = 0.166, *t* = 1.432, df = 23.26; Fig. 1*D*). These data together indicated that FLP-18 functions through the G-protein coupled receptors NPR-1 and NPR-4 to control reversal length.

NPR-1 and the *Brugia malayi* homolog of NPR-4 have been shown to have inhibitory functions through FLP-18 (Frooninckx et al., 2012; Anderson et al., 2014). Hence, it is likely that FLP-18 functions through the NPR-4 and NPR-1 receptors and allows for modulation of the AVA and ASE neurons. Together, these results indicate that FLP-18 could modulate the activity of AVA and ASE sensory neuron through the NPR-4 and NPR-1 receptors and this modulation allows the animal to control reversal lengths. Although our experiments point to the fact that NPR-4 is functioning in the AVA neuron and NPR-1 functions in the ASE neurons, the fact that expressing NPR-1 in other sensory neurons and/or RMG neurons (with *gpa-3* and *flp-5* promoters) also allows for rescue of the increased reversal phenotype seen in the *npr-1* mutants indicates the possibility of NPR-1 functioning in other neurons to maintain reversal length. Further analysis at the level of single neurons could allow one to understand whether NPR-1 has a much more complex role in the process of regulating reversal length.

### Mutants in *flp-18* show increased AVA activity

**Figure 2.**
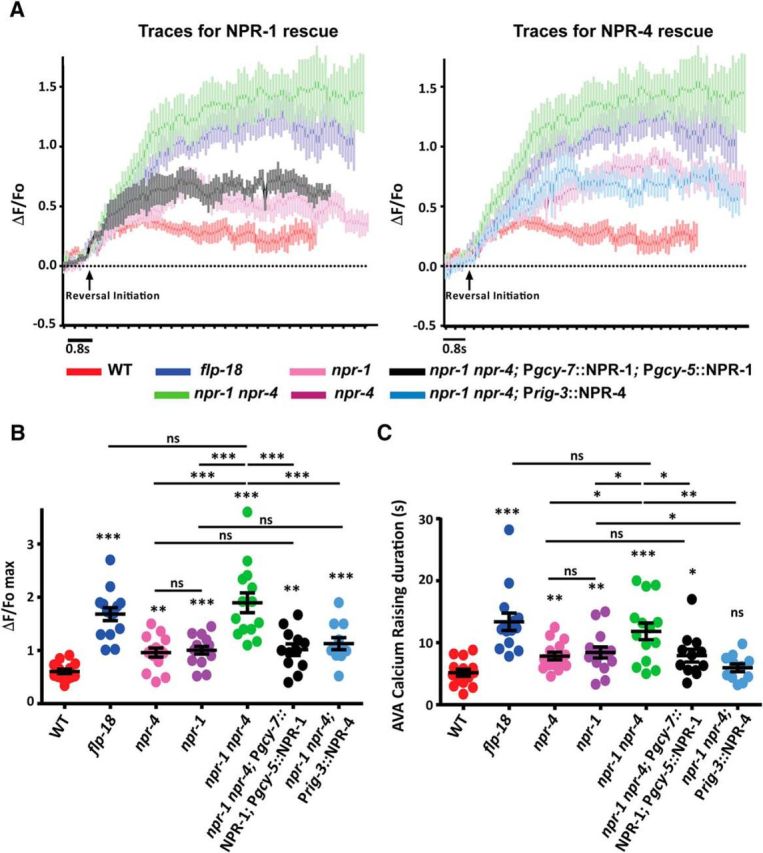
Mutants in *flp-18* and its receptors *npr-1* and *npr-4* show increased AVA activity. ***A***, Comparison of the mean values for calcium transients in AVA during a single reversal event WT *C. elegans*, *flp-18* mutants, *npr-1 npr-4* double mutants, and NPR-1 rescue (left) or NPR-4 rescue lines (right). Dark solid line and light color shading represent mean ± SEM; *n* = 12–14 animals for each genotype. Arrow indicates the reversal initiation point. ***B***, Maximum calcium transients in the AVA neuron. The calcium peaks were compared for WT animals, *flp-18* mutants, *npr-4* mutants, *npr-1* mutants, *npr-1 npr-4* double mutants, NPR-1 rescue in ASE neurons, and NPR-4 rescue in the AVA neuron (*n* = 12–14). ***C***, Comparison of calcium-raising duration in AVA during reversal in WT, *flp-18* mutants, *npr-1* mutants, *npr-4* mutants, *npr-1 npr-4* double mutants, NPR-1 rescue in ASE neurons, and NPR-4 rescue in the AVA neuron (*n* = 12–14). *p < 0.05, **p < 0.01, ***p < 0.001.

Previous studies have shown an increase in calcium levels in the AVA command interneuron during spontaneous reversals (Ben Arous et al., 2010; Piggott et al., 2011; M. Zheng et al., 2012). Further, Gray et al. (2005) have shown that *C. elegans* with laser-ablated AVA neurons do not show longer reversals during local search behavior. This information, along with our previously described results where NPR-4 expression in AVA rescues the *npr-4* mutant phenotype, indicates a possible role of AVA in controlling the length of backward movement. How activity of AVA is related to length of reversals is not clear. We reasoned that calcium-raising duration and/or calcium levels of AVA during reversals might have some relation with the length of reversals. To delineate the relation of AVA activity with length of reversals, we used *C. elegans* with GCaMP5.0 expressed in the AVA interneuron using the *rig-3* promoter (Larsch et al., 2013) and measured calcium levels in freely navigating animals during spontaneous reversals. Consistent with our previous results, *flp-18* mutants showed increased reversal length compared with WT animals (Fig. 2). Furthermore, very strikingly, *flp-18* mutants showed an increase in calcium levels (*p* < 0.0001, *t* = 8.336, df = 16.13) and in the calcium-raising duration for AVA during reversals (*p* < 0.0001, *t* = 5.464, df = 17.22; Fig. 2*A–C*; https://www.amazon.com/clouddrive/share/oEQEG4UJTiZxidtYTldE2AKcgppXtZgp5luQd7RJYWD and https://www.amazon.com/clouddrive/share/2xZpGLfjhSBcfbe4GAFZVDdQM6TRdo1CrZ4NvbUeoMp).

Our results show that FLP-18 functions through NPR-4 in AVA and NPR-1 in ASE to control reversal length (Fig. 1*A*,*B*). Both *npr-4* and *npr-1* mutants showed an increase in calcium levels: WT and *npr-1* (*p* < 0.0001, *t* = 4.656, df = 20.78), WT and *npr-4* (*p* = 0.0015, *t* = 3.712, df = 19.11), and calcium-raising duration of AVA during reversal: WT and *npr-1* (*p* = 0.0054, *t* = 3.085, df = 22.06), WT and *npr-4* (*p* = 0.0036, *t* = 3.198, df = 25.8), compared with WT control animals (Fig. 2). Increased AVA activity in *npr-4* mutants suggests that FLP-18 released from AVA and other neurons controls the activity of AVA through the NPR-4 receptor. The function of FLP-18 through NPR-4 in AVA provides a novel example of self-regulation of neuronal activity by neuropeptide signaling. NPR-4 has been reported to be involved in various functions in *C. elegans*, but the signaling mechanism underlying NPR-4 function is not completely clear. Increased activity of AVA in *npr-4* mutants during reversals suggests an inhibitory function of the NPR-4 receptor. Our previous results show that NPR-1 functions in ASE to control reversal length (Fig. 1*A*,*C*). NPR-1 has been shown to function through inhibitory signaling (Cheung et al., 2005; A. J. Chang et al., 2006; Rogers et al., 2006). Hence, *npr-1* mutants might have increased activity in ASE sensory neurons. Increased activity of AVA during reversals in *npr-1* mutants could be due to ASE-mediated change in the activity of AIB and AWC neurons, which are known to play important roles in modulating reversal behavior (Gray et al., 2005; Chalasani et al., 2007; Piggott et al., 2011). Changes in the activity of AIB and AWC could in turn affect the AVA interneuron behavior.

Independently, both *npr-1* and *npr-4* mutants have increased calcium levels and calcium-raising duration of AVA compared with WT animals but show significantly lower levels compared with *flp-18* mutants: calcium levels *flp-18* and *npr-1* (*p* = 0.0001, *t* = 4.764, df = 21.43), *flp-18* and *npr-4* (*p* = 0.0001, *t* = 4.858, df = 23.26; Figure 2*A–C*). To test whether increased calcium levels and calcium-raising duration of AVA in *flp-18* mutants are due to both NPR-4 and NPR-1, we measured the calcium levels in *npr-1 npr-4* double mutants. Similar to *flp-18* mutants, *npr-1 npr-4* double mutants showed longer reversals with increased calcium levels: WT and *npr-1 npr-4* (*p* < 0.0001, *t* = 6.772, df = 14.37), n*pr-1* and *npr-1 npr-4* (*p* = 0.0003, *t* = 4.467, df = 17.02), n*pr-4* and *npr-1 npr-4* (*p* = 0.0002, *t* = 4.582, df = 18.25); and calcium-raising time of AVA: WT and *npr-1 npr-4* (*p* = 0.0003, *t* = 4.533, df = 17.43), n*pr-1* and *npr-1 npr-4* (*p* = 0.0476, *t* = 2.096, df = 22.48), n*pr-4* and *npr-1 npr-4* (*p* = 0.0157, *t* = 2.664, df = 18.22) compared with WT, *npr-4*, or *npr-1* animals (Fig. 2*A–C*; https://www.amazon.com/clouddrive/share/STttKu2n4pkvrKDnHghDGUD0d3nEoPtw5YxNaxaz7T1). This increase could be due to the synchronized effect of independently increased activity of AVA due to loss of *npr-4* and ASE due to loss of *npr-1*. Finally, we were able to rescue the *npr-1* phenotype seen in the *npr-1 npr-4* double mutants by expressing NPR-1 in the ASE neurons. As can be seen from the traces, the rescued line shows a calcium level (*p* = 0.696, *t* = 0.396, df = 21.71) and calcium-raising time of AVA (*p* = 0.956, *t* = 0.0556, df = 18.51) similar to that seen in *npr-4* mutants (Fig. 2*A–C*). We were also able to rescue the *npr-4* mutant phenotype seen in the double mutants by expressing NPR-4 specifically in the AVA neuron. The rescued line shows similar calcium levels (*p* = 0.364, *t* = 0.932 df = 17.84) and calcium-raising time of AVA (*p* = 0.0343, *t* = 2.257 df = 21.94) as seen in the *npr-1* mutants (Fig. 2*A–C*). These results show that NPR-1 expression in the ASE sensory neurons and NPR-4 expression in the AVA command interneuron are able to rescue the increased activity of the AVA neuron, seen in the *npr-1 npr-4* mutant *C. elegans*.

### CREB1/CRH-1 regulates FLP-18 levels

**Figure 3.**
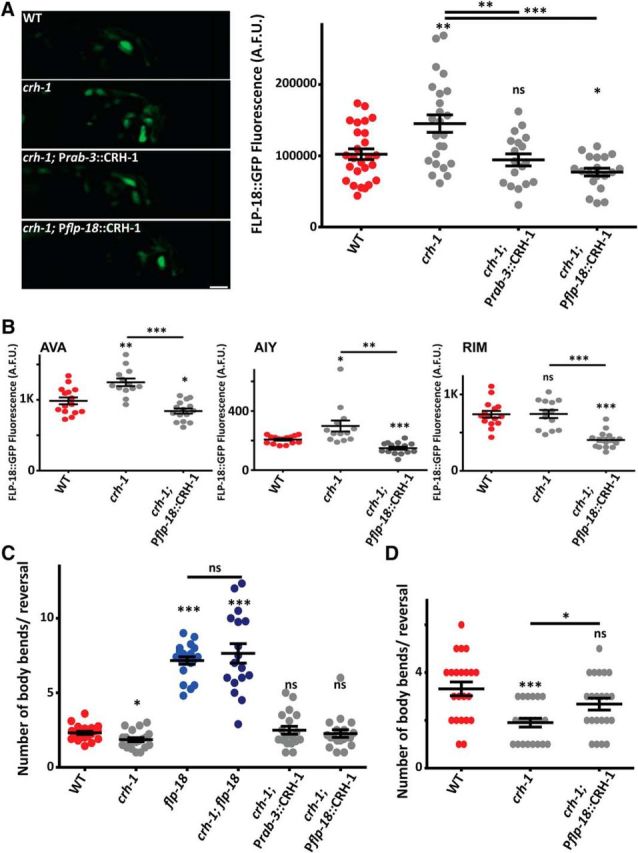
CREB1/CRH-1 regulates FLP-18 expression. ***A***, Quantitation of P*flp-18*::FLP-18::*sl2*::GFP in WT, *creb1/crh-1*, and CREB1/CRH-1f rescue lines (*n* = 20). Scale bar, 10 μm. Right, *crh-1; Prab-3::*CRH-1f and *crh-;1 Pflp-18::*CRH-1f are compared with WT control animals (ns, *) and to crh-1 mutants (indicated by lines drawn above the graph with respect to the *crh-1* plot). ***B***, P*flp-18*::FLP-18::*sl2*::GFP fluorescence from the specific neurons AVA, AIY, and RIM from WT, *crh-1* mutants, and *crh-1* mutants expressing CRH-1f under the *flp-18* promoter (*n* = 15). ***C***, Body bends per reversal in WT, *creb1/crh-1, flp-18, creb1/crh-1; flp-18* double mutants and CREB1/CRH-1f rescue lines (*n* = 20). ***D***, Body bends per reversal upon ChR2-based activation of ASH interneurons in WT, *creb1/crh-1*, and rescue of the *creb1/crh-1* mutant phenotype by expressing CRH-1f under the *flp-18* promoter (*n* = 20). *p < 0.05, **p < 0.01, ***p < 0.001.

Having found FLP-18 to be required for controlling reverse body bends, we reasoned that changes in the cellular levels of FLP-18 could change the length of reversals, and hence food search strategy in response to different environmental conditions. We next asked how FLP-18 expression could be regulated in *C. elegans*. On analyzing the promoter sequence of FLP-18, we found multiple cAMP response element (CRE) sites (5′-TGACGTCA-3′): that is, CREB1/CRH-1 binding sites (Craig et al., 2001). Two additional pieces of evidence prompted us to determine whether CREB1/CRH-1 regulated the levels of FLP-18. First, *creb1/crh-1* mutants have been shown to effect reversal length; the mutants show short reversals compared with WT animals in tap response assays (Timbers and Rankin, 2011). Second, there have been two recent reports implicating CREB1/CRH-1 in maintaining the levels of other FMRFamide-like peptides, FLP-6 (Chen et al., 2016) and FLP-19 (Rojo Romanos et al., 2017). To determine whether CREB1/CRH-1 regulates FLP-18 expression, we analyzed the expression of FLP-18 using the P*flp-18*::FLP-18::*sl2*::GFP reporter in WT and *creb1/crh-1* mutant animals. We found a significant increase in the FLP-18 expression in *creb1/crh-1* mutant animals compared with WT control animals (*p* = 0.0049, *t* = 2.982, df = 38.53), where neurons expressing FLP-18 showed increased expression in *creb1/crh-1* mutants (Fig. 3*A*). Because CREB1/CRH-1 seems to act as a regulator of FLP-18 expression, we reasoned that CRH-1f (which has the DNA binding bZIP domain but lacks the N-terminal activating kinase inducible domain; Wormbase gene: WBGene00000793) could be inhibiting FLP-18 expression. To test this hypothesis, we expressed CRH-1f under the pan-neuronal *rab-3* promoter in the *creb1/crh-1* mutant line. We found that pan-neuronally expressing CRH-1f could completely rescue the increased FLP-18 levels seen in *creb1/crh-1* mutant animals (*p* = 0.0015, *t* = 3.411, df = 38.32; Fig. 3*A*). We next asked whether expressing CREB1/CRH-1 in only FLP-18-positive neurons could rescue the increased FLP-18 expression levels. Targeted expression of CRH-1f cDNA under the *flp-18* promoter sequence restored the increased FLP-18 levels seen in the *creb1/crh-1* mutant animals to WT levels (*p* = 0.0103, *t* = 2.687, df = 42.04; Fig. 3*A*). We next went on to get a better understanding of the changes in FLP-18 levels in specific neurons. To do this experiment, we used colocalization markers that would allow us to identify the AVA, AIY, and RIM neurons (see Materials and Methods). We quantitated the GFP expression from these neurons in WT and *creb1/crh-1* mutant backgrounds. Our data indicated that, in *creb1/crh-1* mutants, there was a significant increase in fluorescence in the AVA (*p* = 0.0013, *t* = 3.613, df = 25.2) and AIY (*p* = 0.029, *t* = 3.613, df = 25.2) neurons, whereas no changes were seen in the RIM neuron (*p* = 0.955, *t* = 0.0565, df = 24.57; Fig. 3*B*). We went on to express CREB1/CRH-1 under the *flp-18* promoter in these mutant lines. In all the three neurons, we saw a very significant reduction in P*flp-18*::FLP-18::*sl2*::GFP expression upon expressing CREB1/CRH-1: *crh-1* and *crh-1;* P*flp-18*::CRH-1f AVA (*p* < 0.0001, *t* = 6.297, df = 21.41), AIY (*p* = 0.0016, *t* = 3.925, df = 13.72), RIM (*p* < 0.0001, *t* = 5.692, df = 18.71; Fig. 3*B*). These data indicate that CREB1/CRH-1 could be acting as a repressor of FLP-18 in the neurons that are involved in the reversal behavior in *C. elegans*.

If CREB1/CRH-1 is indeed required to inhibit FLP-18 levels, we hypothesized that *creb1/crh-1* mutants may show differences in reversal lengths compared with WT *C. elegans*. Upon testing the mutants, we found that the mutants show a significant decrease in reverse body bends as would be expected if CREB1/CRH-1 inhibits FLP-18 expression (*p* = 0.0013, *t* = 3.613, df = 25.2; Fig. 3C; https://www.amazon.com/clouddrive/share/FoXcFLU0U3uGnXdGsbo07rorAhQ6ro7dcPcj89zE87B). Again, we could rescue this phenotype by expressing CRH-1f pan-neuronally (*p* = 0.536, *t* = 0.628, df = 24.63) as well as more specifically in FLP-18-expressing neurons (*p* = 0.881, *t* = 0.141, df = 25.25; Fig. 3*C*). Further, double mutants of *creb1/crh-1* and *flp-18* showed a phenotype that was indistinguishable from the *flp-18* single mutants (*p* = 0.4966, *t* = 0.6920, df = 20.82; Fig. 3*C*), again indicating that CREB1/CRH-1 is acting upstream of FLP-18.

We next went on to explore the reversal body bends in *creb1/crh-1* mutants upon activating the ASH neuron (Guo et al., 2009). We found a significant decrease in reverse body bends in these mutants (*p* = 0.0002, *t* = 4.136, df = 35.03), and this phenotype could be rescued by expressing CRH-1f under the *flp-18* promoter (*p* = 0.1037, *t* = 1.664, df = 41.1; Fig. 3*D*).

Together, these results show that CREB1/CRH-1 inhibits FLP-18 expression in the *C. elegans* nervous system.

### Starvation affects reverse body bends through increase in FLP-18 levels

**Figure 4.**
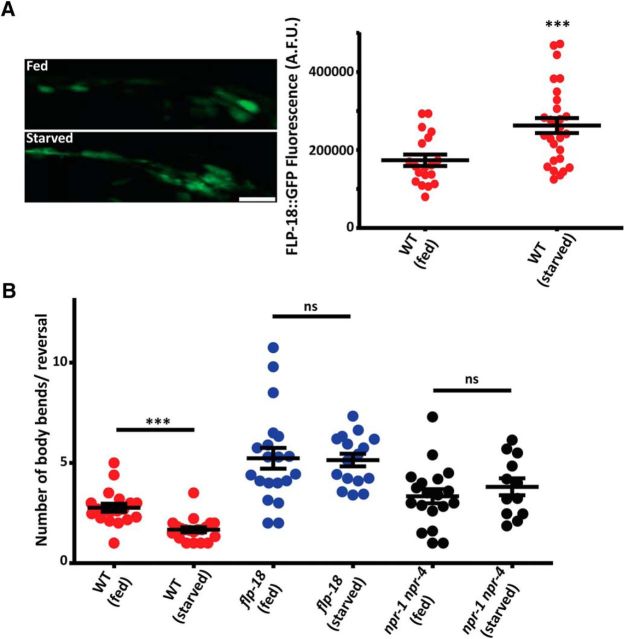
Starvation causes increased FLP-18 levels in *C. elegans*. ***A***, P*flp-18*::FLP-18::*sl2*::GFP expression in WT animals that are fed or starved for 24 h (*n* = 20). Scale bar, 10 μm. ***B***, Body bends per reversal in WT (fed and starved for 24 h), *flp-18* (fed and starved for 24 h), and *npr-1 npr-4* (fed and starved for 24 h) (*n* = 20). ***p < 0.001.

Change in locomotion is required for effective exploration of the environment under different physiological conditions. Modulating reversal length is important to allow *C. elegans* to switch their navigational strategy during prolonged starvation. Our results thus far indicate that reversal length is affected by levels of FLP-18. This prompted us to investigate the expression of FLP-18 during starvation. To study the effect of starvation on FLP-18 levels, we starved the *C. elegans* for 24 h and extracted RNA from fed and starved animals. We estimated the levels of *flp-18* transcripts through qPCR under both conditions and found that there was a 20-fold increase in FLP-18 levels upon starvation (data not shown). We also validated these data by quantitating the levels of P*flp-18*::FLP-18::*sl2*::GFP in well-fed or 24 h starved *C. elegans*. Again, we found an increase in P*flp-18*::FLP-18::*sl2*::GFP levels upon starvation (*p* = 0.0006, *t* = 3.691, df = 44.91; Fig. 4*A*). These two pieces of data indicate that starvation causes an increase in FLP-18 levels.

We next went on to ask whether the increase in FLP-18 levels could cause changes in reversal body bends. Upon analyzing the reversal lengths in fed *C. elegans* and animals starved for 24 h, we saw a significant decrease in the number of body bends in starved animals during each reversal (*p* < 0.0001, *t* = 4.619, df = 30.71; Fig. 4*B*; https://www.amazon.com/clouddrive/share/jErueH1BjmPVhpLuixNKGpG0XTYMQifULeVn0x6hY0r and https://www.amazon.com/clouddrive/share/4nKmmo0ps2EK7JIMYCaH63unAACD5tmNlasb9rJDf3t). If the decreased reversal body bends is indeed due to increased FLP-18 levels, then loss of FLP-18 should abolish the decrease in body bends that we see in starved WT animals. We went on to analyze the reverse body bends in *flp-18* mutants that were starved for 24 h along with control animals that were not starved. We found that the decrease in body bends per reversal that is seen in WT animals upon starvation is completely abolished in *flp-18* mutant animals (*p* = 0.888, *t* = 0.142, df = 30.22; Fig. 4*B*; https://www.amazon.com/clouddrive/share/RKqSKIVBWrVFLsqiAmL2cgwxcI92mV2JfrGRsQfp6Sd and https://www.amazon.com/clouddrive/share/ry5teKQOs5vNsYdp6WTnATS5S9c7kqhzg4Wkdl5kB7X). These data indicate that FLP-18 levels are regulated in *C. elegans* during starvation. Starved animals show increased FLP-18 expression; and consistent with the fact that increased FLP-18 levels could cause shorter reversals, analysis of these *C. elegans* showed that they indeed made shorter reversals.

We next asked whether FLP-18 is functioning through NPR-1 and NPR-4 during starvation. To do this, we went on to look at the reversal length in the *npr-1 npr-4* double mutants in animals that were either fed or starved for 24 h. Again, we found that the *npr-1 npr-4* mutants behaved like the *flp-18* mutants and had lost the ability to reduce reversal length as was seen in WT animals (*p* = 0.4001, *t* = 0.856, df = 24.13; Fig. 4*B*). Together, these experiments show that the FLP-18 pathway functioning through NPR-1 and NPR-4 is required for reducing the reversal length during starvation in *C. elegans*.

One discrepancy that we found in these experiments was that there was a significant reduction in reversal length in *flp-18* mutants compared with our previous reversal data. Further, the *npr-1 npr-4* double mutants appear to have a lower reversal length than *flp-18* mutants at 24 h fed and starved conditions (Fig. 1*A* vs Fig. 4*B* for *flp-18* and *npr-1 npr-4*). One possible reason could be the age of the animals that differs in both sets of experiments. At 78 h of development, which is when the later experiments were performed, all the animals have multiple eggs. This is not the case for the initial data, which were done at 54 h after egg-laying (including hatching) where the animals had no/few eggs (Chiba and Rankin, 1990). Although the starvation data implicate *flp-18* and *npr-1 npr-4* to be involved in modulating reversal length during starvation, more experiments would be required to pinpoint the exact mechanism of how these molecules function during satiety and starvation.

## Discussion

Neuromodulators modify behaviors by shaping the properties of neural circuits. Here, we reveal the role of the neuropeptide FLP-18 in regulating reversal length in *C. elegans*. Our initial observations showed that *flp-18*-null mutant animals make longer reversals compared with WT control animals. Reversal length has been shown to be positively correlated with the probability of change in direction of *C. elegans* movement after reversal (Gray et al., 2005). Hence, reversal length is especially important for behaviors, such as chemotaxis and pathogen avoidance, which require the animals to change locomotory strategies to move toward the source of food or away from a pathogen (H. Luo et al., 2013). Similarly, the switch that *C. elegans* make from local search to global search after prolonged starvation also depends upon the regulation of reversal length. Our study identifies FLP-18 as a key molecule in the regulation of reversal length under different genetic and environmental conditions. Null mutants of *creb1/crh-1* show shorter reversal length (Fig. 3*A*) and low chemotaxis indices (Y.D., unpublished data) consistent with defects in the ability of the mutants to change direction in response to chemical gradients. We show that FLP-18 expression is negatively regulated by the transcription factor CREB1/CRH-1. Further, *flp-18; crh-1* double mutants show behaviors similar to *flp-18*-null mutants, indicating that FLP-18 is likely to be functioning downstream of CREB1/CRH-1. We further showed that, during starvation, WT *C. elegans* tend to make shorter reversals, which in turn may result in a change in the animal's exploratory strategy from local search to global search behaviors (Gray et al., 2005; current study). Our experiments suggest that this decrease in the reversal length could be due to increased levels of FLP-18. This is further corroborated by the fact that *flp-18*-null mutants are unable to regulate reversal length under our assay conditions (Fig. 4).

**Figure 5.**
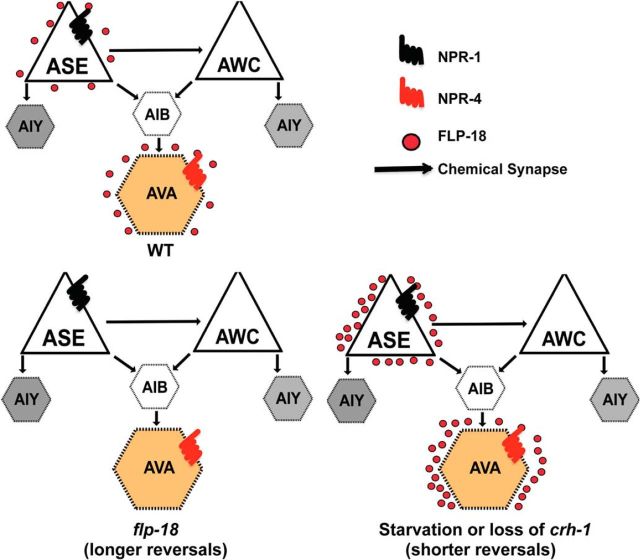
Schematic model for the neuropeptide FLP-18 based modulation of the reversal circuit at the level of sensory neurons and interneurons. In the absence of FLP-18, the reversals are longer and, during starvation, there is more FLP-18 and shorter reversals. AVA and AIY are not the only neurons that express FLP-18; other neurons, such as RIM and RIG, also express FLP-18, although they are not shown in the illustration.

Reversal circuit analysis revealed that NPR-1, NPR-4, and NPR-5 could be the potential candidates affecting reversal behavior through FLP-18 (Li and Kim, 2014). Mutants in *npr-4* show significant increase in reversal length compared with WT animals but significantly less than that seen in *flp-18* mutants, which suggests that NPR-4 is not the only receptor through which FLP-18 is modulating the reversal circuit. Reversal behavior analysis of *npr-1* mutants also showed a similar behavior to *npr-4* mutants, whereas *npr-1 npr-4* double mutants showed a significant increase in reversal length comparable with that seen in *flp-18* mutants. Behavioral analysis showed that NPR-5, which is expressed in sensory neurons and the first layer of interneurons, is not involved in this process. To further elaborate the site of action of FLP-18, we performed neuron-specific rescue experiments. Our rescue experiments suggest that NPR-4 functions in AVA whereas NPR-1 could function in the ASE sensory neurons. A possible circuit could involve NPR-1 functioning in the ASE sensory neuron to mediate changes in the AVA command interneuron through the AIB interneurons and/or the AWC sensory neurons, at the same time NPR-4 could affect the AVA neuron directly, which is also one of the neurons that expresses FLP-18, thereby allowing for a possible mechanism for the AVA neuron to modulate its own activity during reversals (illustrated in Fig. 5). However, it is plausible that NPR-1 could function in other sensory neurons or interneurons to allow for maintaining reversal length. Our data suggest that FLP-18 modulates the activity of reversal circuits at command interneuron and sensory neuron levels simultaneously. Finally, our data also suggest that shorter reversals during starvation could be mediated by the FLP-18/NPR-1/4 signaling pathway (Fig. 4*B*; illustrated in Fig. 5).

We also studied the reversal circuit through calcium imaging during exploratory behavior. Calcium imaging of AVA in *flp-18* mutants showed similar patterns as observed in *npr-1 npr-4* double mutants. Surprisingly, calcium levels of AVA during reversals in *npr-1* and *npr-4* mutants were comparable and significantly lower than *flp-18* and *npr-1 npr-4* mutants (Fig. 2). These results suggest two important points. First, FLP-18 functions through NPR-1 and NPR-4 supporting our behavioral experiments. Second, the FLP-18/NPR-1/4 system appears to modulate the reversal circuit through inhibitory signaling. Together, these observations point to a possible role of FLP-18 in modulating reversal length by regulating the duration of activity and calcium levels of AVA through NPR-4 and NPR-1. Our work provides molecular insights into the modulation of the locomotory circuit by showing that the FLP-18/NPR-1/NPR-4 signaling pathway plays a critical role in modulating locomotion-based behaviors under various genetic and environmental conditions.
