# Supplementary figures and images for: FLP-18 Functions through the G-Protein-Coupled Receptors NPR-1 and NPR-4 to Modulate Reversal Length in Caenorhabditis elegans
Source: J Neurosci. 2018 May 16;38(20):4641–54. doi: 10.1523/JNEUROSCI.1955-17.2018 (PMC5965667; doi:10.1523/JNEUROSCI.1955-17.2018)

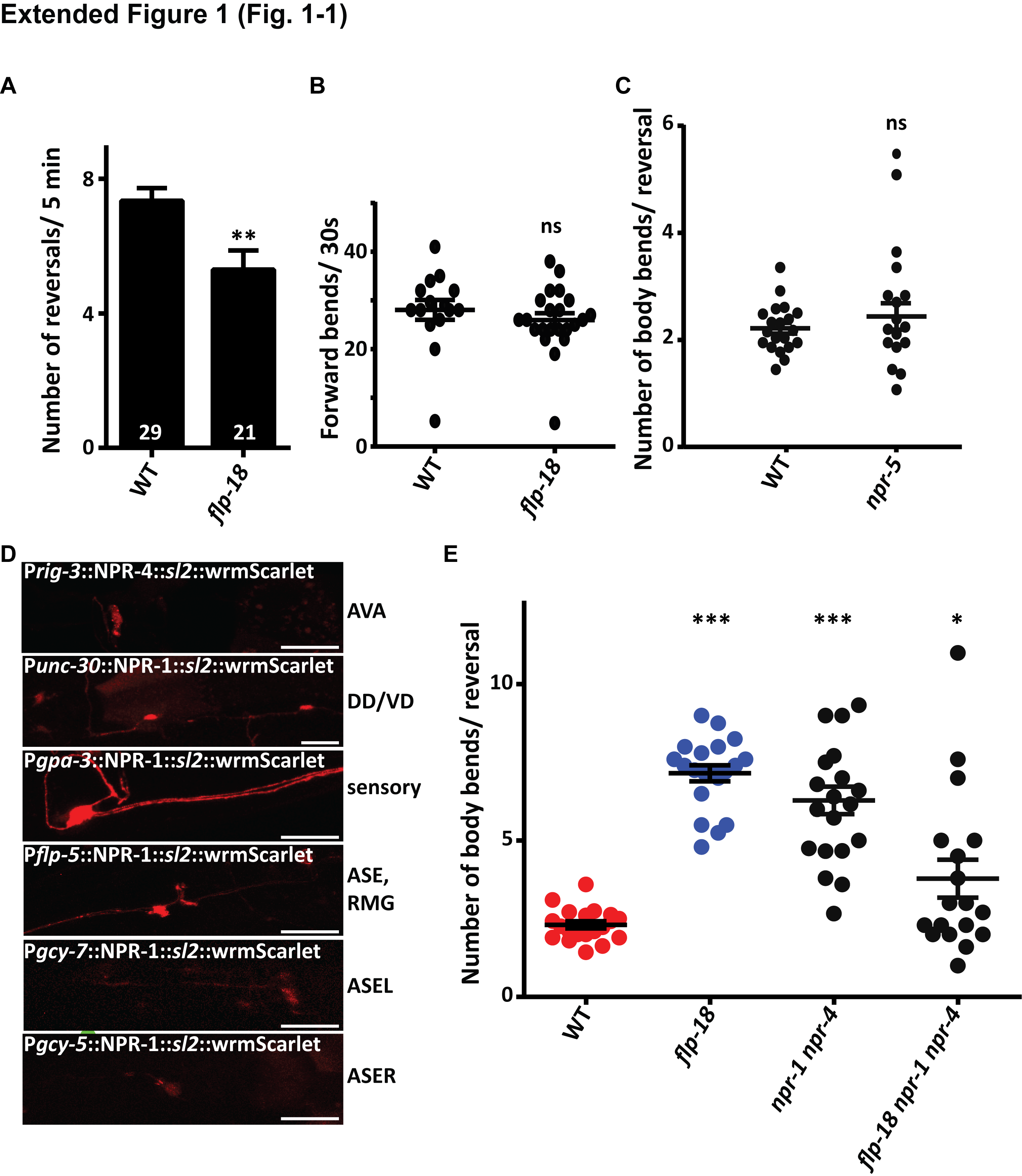

Supplement: Figure 1-1 [file zns999180772so1.tif]
